# Bioethanol Production from Fermentable Sugar Juice

**DOI:** 10.1155/2014/957102

**Published:** 2014-03-12

**Authors:** Hossain Zabed, Golam Faruq, Jaya Narayan Sahu, Mohd Sofian Azirun, Rosli Hashim, Amru Nasrulhaq Boyce

**Affiliations:** ^1^Institute of Biological Sciences, University of Malaya, 50603 Kuala Lumpur, Malaysia; ^2^Department of Petroleum and Chemical Engineering, Faculty of Engineering, Institut Teknologi Brunei, Tungku Gadong, P.O. Box 2909, Brunei Darussalam

## Abstract

Bioethanol production from renewable sources to be used in transportation is now an increasing demand worldwide due to continuous depletion of fossil fuels, economic and political crises, and growing concern on environmental safety. Mainly, three types of raw materials, that is, sugar juice, starchy crops, and lignocellulosic materials, are being used for this purpose. This paper will investigate ethanol production from free sugar containing juices obtained from some energy crops such as sugarcane, sugar beet, and sweet sorghum that are the most attractive choice because of their cost-effectiveness and feasibility to use. Three types of fermentation process (batch, fed-batch, and continuous) are employed in ethanol production from these sugar juices. The most common microorganism used in fermentation from its history is the yeast, especially, *Saccharomyces cerevisiae*, though the bacterial species *Zymomonas mobilis* is also potentially used nowadays for this purpose. A number of factors related to the fermentation greatly influences the process and their optimization is the key point for efficient ethanol production from these feedstocks.

## 1. Introduction

Energy crisis is a growing global concern nowadays because of the dependence on petroleum-based fossil fuel which is exhausted very fast to meet the continuously increasing demands. Besides, fossil energy also has the direct impact on the atmosphere [1]. It has been realized that fossil energy causes greenhouse gas emissions that have adverse effects on the environment. Burning of petroleum-based fuels causes the increase of CO_2_ level in the environment which is directly responsible for global warming [2]. Another important concern of fossil fuel reliance is the political crisis. For example, incidence of oil supply disruption by the Middle East countries in the 1970s caused unrest in this essential sector [3, 4]. Consequently, it is an ongoing interest to find out a renewable and environmentally friendly source of energy for our industrial economies and consumer societies [5]. Bioethanol in this aspect is an attractive option for renewable and sustainable energy source.

Among the advantageous properties of bioethanol as fuel energy, higher octane number (108), evaporation enthalpy, and flame speed and wider range of flammability are worth mentioning. Due to these characteristics, fuel ethanol gives higher compression ratio (CR) with shorter burning time, eventually providing a better theoretical efficiency than that of gasoline in an integrated circuit (IC) engine [6]. Besides, it can be used as transportation fuel in various feasible ways, directly or blend with gasoline called “gasohol.” The most common blended bioethanol used in USA is E-10 containing a concentration of 10% ethanol and 90% gasoline [7]. Brazil, on the other hand, uses pure ethanol or blended ethanol in a combination of 24% ethanol with 76% gasoline [8]. Furthermore, a 5% of bioethanol blended with petrol can be used under the EU quality standard-EN/228 without any modification of engines, whereas, to use higher concentration of this fuel, namely, E-85 (85% ethanol), engine modification is required [9]. Bioethanol, in another aspect, is an environmentally friendly oxygenated fuel containing 35% oxygen which is suitable to keep down the emission of particulate and nitrogen oxides as well as other greenhouse gases during combustion [10, 11]. Moreover, due to having lower ambient photochemical reactivity, it reduces the interference on ozone [12]. This fuel energy is also a safer substitute to methyl tertiary-butyl ether (MTBE), a common additive used in gasoline for clean combustion [13].

However, for sustainability and economic viability, it is important to give concentration in cheaper ethanol production so that this fuel can compete with petroleum [14, 15]. Currently, industrial bioethanol production plants employ mainly two types of primary feedstocks such as starch from cereal crops and juice or molasses from sugar crops [7, 16, 17]. About 60% of the global ethanol is produced from sugar crops, while the remaining 40% is produced from starchy grains [18]. Bioethanol from lignocellulosic biomass has recently been studied extensively but still it is confined to the laboratory or pilot plant. It is easier and cheaper to use free sugar containing juice as feedstock of ethanol than starch or lignocellulosic biomass due to the nonrequirement of costly steps such as pretreatment and/or hydrolysis to get fermentable sugars [19–22]. This paper will deal with the investigative works on bioethanol production from sugar juices obtained from several energy crops along with fermentation technology, microorganisms used, and the influencing parameters on the process.

## 2. Potential Juices Used as Feedstocks

Bioethanol can be produced directly from the free sugar containing juices of some crops, converting sucrose or monosaccharides, especially, glucose, into ethanol via fermentation with microorganisms [23, 24]. Several potential crops yield free sugar containing juices that are employed in bioethanol production either in laboratory or commercial scale (Table 1). Sugarcane, sugar beet, sweet sorghum, and some fruits are the good sources of sugar-rich juices used as feedstocks in ethanol production [25]. Direct fermentable juices obtained from these crops contain free sugars, especially, sucrose, glucose, and fructose [26], that make them more cost-effective feedstocks in fuel ethanol industry than starchy or lignocellulosic materials [21, 25] (Table 2). Sucrose which is the major sugar in fermentable juices is readily broken down into glucose and fructose during earlier stage of fermentation by invertase enzyme, indigenously found in the periplasmic space of yeast used in the process [27]. In a general procedure, juice is obtained from sugar crops, supplemented with ammonium sulfate or other nitrogen sources, sterilized, with pH and sugar concentration being adjusted, and then fermented using microorganisms, especially, yeast, under a suitable condition [28]. The main disadvantages of using juice as feedstocks are the low storability and microbial decomposition. Dodić et al. [27] studied beet juice and reported that thick juice could be concentrated to a high sugar content giving reduced storage volume and ultimately lower microbial inhibition as compared with thin juice. During processing of juices, conventional liming-carbonation method is applied in the industry which is an energy consuming process and responsible for production of waste and CO_2_. Use of membrane technology to purify sugar juices can efficiently replace this traditional process [29]. In addition, several investigators also reported that membrane filtration of sugar juice could give higher purity, that is, higher sucrose concentration as compared to the conventional liming-carbonation method [30–33].

Sugarcane (*Saccharum officinarum*) is a C4 plant having high capability to convert solar radiation into biomass [34]. It is the most important feedstock grown in tropical and subtropical countries that can be used as juice or molasses (by product of sugar mills) for fuel ethanol production. Total fermentable sugar content in sugarcane juice is about 12–17% in which 90% these sugar is sucrose and the remaining 10% is glucose and fructose [35]. Sugar content in juice varies based on variety, maturity, and harvest time [26]. Sugarcane juice contains adequate amount of organic nutrients and minerals in addition to free sugars making it an ideal raw material for bioethanol production. It is used as the main feedstock for ethanol production in Brazil [16, 36], whereas molasses is the principal feedstock in India [37].

Sugar beet (*Beta vulgaris*) and its industrial byproduct (beet molasses) are important feedstocks for bioethanol production because of their free sugar contents that can be employed for fermentation without any modification. Raw beet juice contains 15–20% of dry matters with 85–90% of fermentable sugars and 10–15% of nonsugars [38]. Due to these available free sugar contents, beet juice can readily be used in fermentation after adjusting its pH making it more profitable feedstock for fuel ethanol production [27]. Potentiality of ethanol production from different intermediates and by products of sugar beet processing such as raw juice [39], thick juice [40], molasses [41], and beet pulp [42] have been studied.

Sweet sorghum (*Sorghum bicolor *L.) is a C4 plant, similar to sugarcane, which is a potential energy crop due to its unique characteristics of high carbon assimilation (50 g/m^2^ per day) and ability to store high levels of extractable sugars in the stalks [21, 43]. Besides, it is marked for high photosynthetic efficiency and can be cultivated in almost all temperate and tropical climate areas in both irrigated and nonirrigated lands [44]. In addition, sweet sorghum has some advantages over other sugar crops as the feedstock for ethanol production such as short growing period (4-5 months), capability of both drought and cold temperature tolerance, and utilizing of both grains and juice for ethanol production [45, 46]. Stalks of sweet sorghum contain soluble free sugars, namely, glucose and sucrose, and insoluble carbohydrates such as cellulose and hemicelluloses [47]. Juice contains approximately 12.5°Bx (degree brix) of sugar at the beginning of the harvest with an increase to approximately 17°Bx during the plant's maturation giving an average value of 15°Bx [48]. Energy cost of bioethanol manufacturing from sweet sorghum juice might be lower as compared to sugarcane or sugar beet juices because this crop production needs lower fertilizer and nitrogen and not require the prefermentation processing [43]. Therefore, considering the potentiality of sweet sorghum for energy and industry, it is one of the most promising ethanol producing crops [49].

## 3. Microorganisms

Involvement of microorganisms in fermentation of sugars is a crucial part of bioethanol production. Some microorganisms have the ability to use glucose in the absence of oxygen for their energy, producing ethanol and carbon dioxide [50, 51]. This property makes them potential bioagents in fermentation technology from the beginning of its history. Sugar fermentation using single cell microorganism, that is, yeast, is one of the oldest practices in biotechnology, widely used for the production of drinking alcohol, namely, beer and wine, in the past time, while, nowadays, this practice is industrially used to produce fuel ethanol from renewable energy sources [52]. Major characteristics of ethanologenic microorganisms to be employed in industrial plants are higher ethanol yield (>90.0% theoretical yield), tolerance to ethanol (>40.0 g/L), good ethanol productivity (>1.0 g/L/h), good growth in simple and inexpensive media, capability of growth in undiluted fermentation broth with resistance to inhibitors, and ability to retard contaminants from growth condition, for example, acidic pH or higher temperature [53].

Some microorganisms such as dried yeast or* Saccharomyces cerevisiae* [54–57],* S. diastaticus* [58],* Kluyveromyces marxianus* [59, 60]*, Pichia kudriavzevii* [26],* Escherichia coli* strain KO11 and* Klebsiella oxytoca* strain P2 [61], and* Zymomonas mobilis* [62–65] have been studied for ethanol production from sugar juices. Among these ethanol producing microorganisms,* S. cerevisiae* is the most attractive choice in fermentation due to its greater efficiency in sugar conversion to alcohol and capability of producing flocs during growth, making it easier to settle or suspend on need [52], and high tolerance to ethanol [66]. Moreover, fermentation of some crop juices containing sucrose employs this yeast for its ability to hydrolyze sucrose into glucose and fructose with invertase enzyme. But the optimum temperature range of* S. cerevisiae *used for ethanol production is 30–35°C that leads the researchers to search for thermotolerant microorganisms. Dhaliwal et al. [26] isolated a strain of thermotolerant yeast (*P. kudriavzevii*) from sugarcane juice and adapted the cells to galactose that produced more ethanol than the nonadapted cells at 40°C.

*Z. mobilis*, a Gram-negative bacterium, is also extensively studied over the last three decades in fuel ethanol production from grains, raw sugar, sugarcane juice, and syrup due to its ethanol tolerance and higher glucose uptake as well as good ethanol production capability [64, 67, 68]. It can produce ethanol from glucose through Entner-Doudoroff pathway using the enzymes pyruvate decarboxylase and alcohol dehydrogenase [69]. Higher ethanol yield (97.0%) and productivity of* Z. mobilis* were reported due to the production of less biomass and maintenance of higher rate of glucose metabolism through its ED pathway, while with* S. cerevisiae *ethanol yield was only 90.0–93.0% [70, 71]. Nevertheless, because of its narrow substrate range, this microorganism cannot immediately replace* S. cerevisiae* in fuel ethanol production.

Culture maintenance is an essential step for effective fermentation. Microorganisms typically employed in fermentation process are heterotrophs that require a carbon and a nitrogen source to grow and survive in the culture media. Without proper media and suitable growth condition, it is difficult to get a healthy inoculum for incorporating microbial cells in fermentation broth. Based on type and strain of microorganisms, their growth condition also varies as mentioned in Table 3.

## 4. Fermentation of Juices

Bioethanol is produced mainly by three types of fermentation, such as batch, fed-batch, or continuous [72] (Table 4). In batch fermentation, feedstock is added to the fermentation vessel along with microorganism, nutrients, and other ingredients at the beginning of fermentation of whole batch followed by recovery of ethanol, while, in fed-batch mode, one or more ingredients are added to the vessel as fermentation is going on [49]. Continuous fermentation involves a constant input of ingredients and removal of output from the fermentation vessel [73]. The selection of most suitable mode of fermentation mainly depends on the kinetics of the microorganisms used and the nature of feedstocks. Batch fermentation is the simple fermentation process due to low cost, less control requirement, easier sterilization, and management of feedstocks as well as employment of unskilled workforce. Besides, most of the ethanol production study from juice feedstocks was carried out by batch fermentation [44, 74]. Fed-batch mode is broadly employed in industrial production due to compiling the benefits from both batch and continuous processes [75]. This mode of fermentation gives some advantages over conventional batch process such as maintenance of maximum viable cell concentration, extended lifespan of cell, higher product accumulation, less inhibitory effect of higher substrate concentration, and control of several critical factors such as pH, temperature, and dissolved oxygen at a specific level through the feedback activities [76–79]. Continuous fermentation that can be carried out in mainly two basic types of reactors; for example, plug flow reactor and continuous stirred tank reactor offer some advantages over batch fermentation. This mode of fermentation needs less downtime for vessel cleaning and filling giving increased productivity with lower cost [80].

Free cells of suitable microorganism are normally used in fermentation that carry out their metabolic function in the fermentation broth producing ethanol from sugars. However, use of immobilized microbial cells on different carriers instead of free cells in fermentation is extensively studied to improve the process which showed some technical and commercial benefits over free cell system due to changes in growth condition, physiological and morphological properties, and catalytic activity of cells [81]. This technique enhances the productivity and ethanol yield and reduces the inhibitory effect of high substrate concentration and product [82–84]. In addition, immobilization prevents cell washout in continuous fermentation that avoids separation or recycle of cells in the process [85]. Several carriers have been reported for cell immobilization including apple pieces [83], k-carrageenan gel, polyacrylamide, g-alumina [86], chrysotile [87], calcium-alginate [88, 89], sugarcane pieces [56], banana leaf sheath [90], and orange peel [91]. Immobilization of* S. cerevisiae* can easily be carried out by enriched cells from culture media and harvested at the log phase of growth followed by entrapping into the carriers [88]. It was reported that* Z. mobilis* in an immobilized cell reactor can produce increased ethanol during fermentation with the capability of tolerating high concentration of sugars [92, 93].

Economic evaluation of fuel ethanol production reveals that more energy is consumed in recovery steps conducted by distillation due to low ethanol concentration in fermented broth [15]. Therefore, increasing the ethanol content in the broth can considerably reduce energy consumption in distillation [94]. Very high gravity (VHG) fermentation is a technique of using high concentration of sugars during fermentation with the output of increased concentration of ethanol. This is a technique employed in fermentation of the processed feedstocks containing 270 g/L or more dissolved solids, that is, free sugars [95, 96]. This technology exploits the enhanced and prolonged growth of microorganism in the presence of low level of oxygen [97] and reduces water consumption, labor cost, and distillation cost with more alcohol production [98]. However, ethanol is a toxic metabolite on yeast cells that may lead to cell lysis and death under this VHG environment with a limited ethanol concentration in the broth. Hence, viability loss of cells should be evaluated during fermentation using methylene blue stain technique or colony forming units (CFU) method [94].

## 5. Impact of Different Factors on Fermentation Ethanol Production

Several factors, especially, temperature, pH, fermentation time, agitation rate, initial sugar concentration, and inoculum size, have an impact on fermentation process as well as ethanol yield.

### 5.1. Temperature

Temperature is an important factor carefully regulated during fermentation as it has vital impact on the process and ethanol production. It was also reported that ethanol production depends on fermentation temperature and to some extent its concentration increases with the increase in temperature [99]. However, high temperature is considered as a stress factor for microorganisms, which is unfavorable for their growth. They produce heat-shock proteins in response to the high temperature and inactivate their ribosomes. In addition, microbial activity and fermentation process are regulated by different enzymes which are also sensitive to high temperature since it denatures their tertiary structure eventually inactivating them [100, 101]. Moreover, microorganisms used in the fermentation process have optimum temperature range for their better growth. Therefore, it is necessary to predetermine an optimum temperature during fermentation for proper microbial growth as well as higher yield of ethanol. It is generally believed that the ideal fermentation temperature range is between 20 and 35°C and high temperature in almost all fermentation processes creates problem [101, 102]. The optimum fermentation temperature for free cells of* S. cerevisiae* is near 30°C [101, 103], while for immobilized cells it is slightly higher probably because they can transfer heat from particle surface to inside the cells [104]. In a study with sweet sorghum juice using immobilized yeast cells, it was reported that at 28°C ethanol yield was 75.79% followed by growing up to the maximum yield (89.89%) at 37°C [104]. In another study with the strain* S. cerevisiae* BY4742 in batch fermentation, Lin et al. [105] reported that the highest specific cell growth rate and specific productivity of ethanol were found at 30–45°C with a significant decrease in cell growth as well as in ethanol yield at 50°C. In case of* Z. mobilis*, the best ethanol concentration (55.57 g/L) was found at 30°C, while the lowest (4.6 g/L) was found at 40°C [64]. Similarly, harmful effect on ethanol concentration using this microorganism was also observed at above 37°C by several investigators [106, 107].

### 5.2. pH

Enhanced ethanol production through fermentation can be obtained by controlling pH of the broth as it is one of the key factors for ethanol production having direct influence on organisms as well as on their cellular processes [108, 109]. In general, H+ concentrations in fermentation broth can change the total charge of plasma membrane affecting the permeability of some essential nutrients into the cells. The optimum pH range for* S. cerevisiae *used in fermentation for ethanol production is 4.0–5.0 [105, 110]. However, very recently, it was reported that this well-known yeast could produce ethanol from date juices even at pH 3.8 [111], though the critical pH for this organism is 2.3 [108]. On the other hand, the highest ethanol yield was obtained using* Z. mobilis* adjusting the pH range of the broth as 5.0–6.0 [112]. Different optimum pH range was also reported for several feedstocks such as 2.8 to 3.4 for sugarcane juice [113] and 4.0 to 4.5 for sucrose [114].

### 5.3. Fermentation Time

Shorter time in fermentation causes inadequate growth of microorganisms eventually causing inefficient fermentation. On the other hand, higher fermentation time causes toxic effect on microbial growth especially in batch mode due to the high concentration of ethanol in the fermented broth. Nadir et al. [115] got the highest ethanol concentrations after 64 h accounting for 40.11 g/L followed by dropping to 37.24 g/L after 72 h fermentation while studying with sweet sorghum. In addition, more time is required to complete fermentation at lower temperature though ethanol yield is the lowest. For example, only 44.0% of sugar was consumed in more than 240 h producing the lowest ethanol when fermentation was carried out at 15°C [116].

### 5.4. Agitation Rate

Agitation plays important role in getting higher yield of ethanol during fermentation by increasing the permeability of nutrients from the fermentation broth to inside the cells and in the same way removing ethanol from the cell interior to the fermentation broth. Agitation also increases the sugar consumption and reduces the inhibition of ethanol on cells. Useful agitation rate is 150–200 rpm for yeast cells in fermentation. Liu and Shen [104] reported the maximum ethanol yield (85.73%) at 200 rpm of agitation. Nevertheless, excess agitation rate is not suitable for smooth ethanol production due to the limited metabolic activities of cells.

### 5.5. Sugar Concentration

Initial sugar concentration is an important influencing parameter as it has the direct effect on fermentation rate and microbial cells. The actual relationship between initial sugar content and the fermentation rate is rather more complex. Generally, fermentation rate will be increased with the increase in sugar concentration up to a certain level. But excessively high sugar concentration will exceed the uptake capacity of the microbial cells leading to a steady rate of fermentation. In batch fermentation, increased ethanol productivity and yield can be obtained at higher initial sugar concentration, but it takes longer fermentation time and subsequently increases the recovery cost. Considering these facts, the optimum sugar concentration in batch fermentation was determined as 24°Bx (equivalent to 190.0 g/L) [44]. Similarly, the optimal ratio of sugar and microorganism concentration was reported as 200.0 g/L and 30.0 g/L, respectively, in an investigation with date juice fermentation [111].

### 5.6. Inoculum Size

Inoculum concentration does not have significant influence on final ethanol concentration but significantly affects sugar consumption rate and ethanol productivity [44]. However, it was reported that ethanol production was increased with the increase in the initial cell numbers from 1 × 10^4^ to 1 × 10^7^ cells/mL and no significant difference in ethanol production was found between 10^7^ and 10^8^ cells/mL. Increased cell concentration within a certain range also reduces fermentation time considerably due to the rapid growth of cells in the fermentation media that immediately consumes fed sugars producing ethanol. Breisha [117] reported that reduction in fermentation time from 72 h to 48 h was found by increasing yeast concentration from 3.0% to 6.0%.

## 6. Conclusion

Although current industrial fermentation for fuel ethanol production employs two types of feedstocks such as free fermentable sugars and starch, free sugars containing juice is more economic than starch feedstocks as the former can directly be used in fermentation without any prior treatment. However, better yield also depends somewhat on the selection of microorganisms and fermentation mode and techniques as well as the influence of several factors. In addition, selection and development of different potential genetic varieties of juice producing crops will also enhance the commercial ethanol production. Several technological advances have already been investigated but most of them are still confined to the laboratory. Therefore, a comprehensive economic and process analysis is required to develop an industrially suitable production strategy that will solve our energy crisis by producing more ethanol in a stable way.

## Figures and Tables

**Table 1:** Different sugar crops investigated for ethanol production using sugar juices as feedstocks.

Name of the crops	Major investigation	Major achievements	Reference
Sugarcane (*Saccharum officinarum*)	Juices were studied (i) without adding supplement, (ii) with addition of 0.5% yeast extract, and (iii) with addition of yeast extract, thiamine, and micronutrients	The highest ethanol concentration (39.4–42.1 g/L) was found in (iii), while the lowest was 11.0 g/L and found in (i)	[61]
	Enrichment technique was applied to isolate a thermotolerant yeast from cane juice for ethanol production at elevated temperature	Isolation and selection of thermotolerant yeast strain that produced high concentrations of ethanol at both 40 and 45°C from the juice	[59]
	Juices were supplemented and cells were adapted to galactose medium	Higher (30.0%) ethanol was found with adapted cells than nonadapted cells	[26]

Sugar beet (*Beta vulgaris*)	Juices were ultrafiltered and were supplemented with mineral salt	Ethanol concentration was found to be 85.0–87.0 g/L	[30]
	Flocculating and nonflocculating yeasts along with *Z. mobilis* were used through immobilization on loofa sponge	The highest ethanol yield (0.44 g/g) with the lowest productivity (0.08 g/h/L) was found by *Z. mobilis *	[3]

Sweet sorghum (*Sorghum bicolor) *	Five different genetic varieties (Keller, BJ 248, SSV84, Wray, and NSSH 104) were investigated for ethanol production	Keller variety produced the highest ethanol (9.0%, w/v)	[118]
	Impact of storage on sugar content loss of juice was studied	Up to 20.0% free sugars lost in 3 days at ambient temperature	[119]

Watermelon (*Citrullus lanatus*)	Juice was used as diluent, additive, and nitrogen source for processed sugar or molasses fermentation	As much as 25.0% w/v sugar was fermented at pH 3.0 (ethanol yield was 0.41–0.46 g/g) or up to 35.0% w/v sugar at pH 5.0 with an ethanol yield of 0.36–0.41 g/g	[120]

Dates (*Phoenix dactylifera*)	Juices were extracted from 3 genetic varieties of dates (Kunta, Bouhatem, and Eguoua) and fermentation was studied at 30°C and natural pH	All varieties produced ethanol with a maximum concentration of 25.0%, v/v	[111]

**Table 2:** Chemical composition of feedstocks derived from different crops.

Feedstocks	Free sugar (%)	Moisture (%)	References
Sugarcane juice	12–17.6	68.0–82.4	[26, 35, 49, 121]
Sugar beet juice	16.5	82.6	[3]
Sweet sorghum juice	16–21.8	78.2	[45, 122]
Watermelon juice	7.0–10.0	90.0–93.0	[120]

**Table 3:** Growth condition of microorganisms involved in ethanol fermentation.

Name of microorganisms (strains/species)	Carbon source (g/L)	Nitrogen source (g/L)	Growth temperature (°C)	pH	Shaking rate (rpm)	Time (h)	Reference
*S. cerevisiae* CICC 1308	Glucose or sucrose (50.0)	Peptone (5.0)	30	5.0	150	48	[104]
*S. diastaticus* Y2416	Maltose (3.0) and glucose (20.0)	Yeast extract (5.0), peptone (5.0)	30	6.0	—	—	[123]
*K. marxianus* DMKU 3-1042	Sugar (50.0–80.0)	Ammonium sulfate (0.5)	35	4.5	170	72	[59]
*P. kudriavzevii* DMKU 3-ET15	Glucose (20.0)	Peptone (20.0)	40	6.5	150	48	[124]
*Z. mobilis *	Glucose (10.0) and sucrose (30.0)	Yeast extract (5.0)	30	6.5	Static	18	[3]
*Z. mobilis* ATCC 10988	Glucose (20.0)	Ammonium sulfate (1.0)	30	6.0	100	24–48	[125]
*E. coli *KO11 and *K. oxytoca *P2	Sucrose (20.0)	Ammonium sulfate (2.0)	30	—	100	24	[61]

**Table 4:** Fermentation process employed in ethanol production from different feedstocks.

Feedstock	Initial sugar (g/L)	Fermentation mode	Processing/techniques	Microorganisms	Temperature (°C)	pH	Agitation rate (rpm)	Time (h)	Ethanol concentration (g/L)	Productivity (g/L/h)	Ethanol yield (%)	Reference
Sugarcane juice	200	Batch	Juice was supplemented with sucrose	*K. marxianus* DMKU 3-1042	40	5.0	300	42–96	67.9	1.42	60.4	[59]
	166	Batch	Recycling of yeast	*P. kudriavzevii *	40	5.5	150	24	71.9	4.00	—	[26]
	173	Repeated batch	Immobilization of cells on sugarcane pieces	*S. cerevisiae *	30	—	—	32	89.73	2.48	—	[56]
	220	Batch	Coculture of immobilized cells on thin-shell silk cocoon	*K. marxianus* DMKU 3-1042 and *S. cerevisiae* M30	37–40	5.0	150	72	77.3–81.4	1.07–1.1	80.23–86.1	[126]
	180	Continuous	Immobilization of cells on chrysotile	Strains of *Saccharomyces *sp.	30	5.0	—	117	13.3–19.4	—	80.4–97.3	[127]
	200	Continuous	Recycling of cells was done in a tower reactor	*S. cerevisiae* IR-2	30	—	130	72	90	18	99	[128]

Sweet sorghum juice	190	Fed-batch	Juice was supplemented with 0.5% ammonium sulphate	*S. cerevisiae* TISTR 5048	30	—	Static	108	116.62–120.28	1.08–1.11	94.12–96.8	[44]
	240–320	Batch	Juice was adjusted with sucrose or molasses for very high gravity fermentation	*S. cerevisiae *NP01	30	4.9	Static	40–72	120.68	2.01	99.8	[96]
	—	Batch	Immobilization on Ca-alginate	*S. cerevisiae CICC 1308 *	37	5.0	200	11	—	—	93.24	[104]

Watermelon juice	183–409	Batch	Juice was supplemented with molasses	Dried yeast	32	3.1–5.0	100	36–160	83.2	—	—	[120]

Sugar beet juice	200	Batch	Immobilization of cells on loofa sponge	*S. cerevisiae*, *Candida brassicae,* and *Z. mobilis *	30	6.5	200	—	—	0.08–0.53	72.4–86.1	[3]
